# The digestion and dietary carbohydrate pathway contains 100% gene mutations enrichment among 117 patients with major depressive disorder

**DOI:** 10.3389/fpsyt.2024.1362612

**Published:** 2024-04-29

**Authors:** Ni Zhang, Jin Li, Zaiquan Dong, Yongbo Hu, Zhihui Zhong, Qiyong Gong, Weihong Kuang

**Affiliations:** ^1^ Department of Psychiatry, West China Hospital, Sichuan University, Chengdu, China; ^2^ Huaxi MR Research Center (HMRRC), Department of Radiology, West China Hospital of Sichuan University, Chengdu, China; ^3^ Laboratory of Non-human Primate Disease Modeling Research, West China Hospital, Sichuan University, Chengdu, Sichuan, China; ^4^ National Clinical Research Center for Geriatrics, West China Hospital, Sichuan University, Chengdu, Sichuan, China National Clinical Research Center for Geriatrics, West China Hospital, Sichuan University, Chengdu, Sichuan, China

**Keywords:** major depressive disorder, carbohydrate pathway, deep sequencing, reactome, gene mutation

## Abstract

**Introduction:**

Major depressive disorder (MDD) is partially inheritable while its mechanism is still uncertain.

**Methods:**

This cross-sectional study focused on gene pathways as a whole rather than polymorphisms of single genes. Deep sequencing and gene enrichment analysis based on pathways in Reactome database were obtained to reveal gene mutations.

**Results:**

A total of 117 patients with MDD and 78 healthy controls were enrolled. The Digestion and Dietary Carbohydrate pathway (Carbohydrate pathway) was determined to contain 100% mutations in patients with MDD and 0 mutation in matched healthy controls.

**Discussion:**

Findings revealed in the current study enable a better understanding of gene pathways mutations status in MDD patients, indicating a possible genetic mechanism of MDD development and a potential diagnostic or therapeutic target.

## 1 Introduction

Major depressive order (MDD) is a psychiatric disorder with high incidence and prevalence. MDD could overwhelmingly compromise patients’ mood, cognitive, occupational and social functions (1). As second leading cause of disability worldwide (2), MDD leads to heavy sociopsychological, physical and economic burdens. The World Health Organization has ranked MDD as the third cause of disease burden by 2008 and the first cause by 2030 (3). Notably, MDD itself induces only less than 40% of total costs, leaving the rest and the largest growing economic burden attributing to comorbid conditions and function restoring (4, 5).

The diagnosis and treatment of MDD has always been challenging in clinical practice (3). One of the most important attributing factors is the lack of thorough understanding of MDD’s mechanisms, somehow making identification and management of this diseases difficult. Even though multiple hypotheses have been proposed to explain MDD pathogenesis, none of them could cover every aspect of MDD (1). Gene/genome analysis has been a major breakthrough point during the past few decades, and an estimated 35% of heritability was reported (1). Polymorphism of single genes, including SLC6A4 (6–8), IKBKE (9), FKBP5 (10), FokI (11), PAWR (12), etc., has been investigated by multiple studies. However, despite that these single genes somehow or to some extent are involved in MDD development or progress, not a single gene was established to be 100% associated with MDD. In the context of genetic research on MDD, the conventional focus on single gene polymorphisms has provided valuable insights into specific genetic contributions. However, a shift towards investigating entire gene pathways offers a more comprehensive understanding of the intricate biological processes involved in MDD. This research strategy allows for the exploration of coordinated gene expressions within pathways, revealing potential synergistic effects that may be overlooked in the study of individual genes. Despite significant strides in understanding the neurobiological aspects and single gene mechanisms of MDD, comprehensive investigations into gene pathways, particularly through deep sequencing techniques, have been limited. This study aims to bridge this gap by employing deep sequencing and gene enrichment analysis based on pathways in the Reactome database.

On the contrary, instead of single gene which works relatively simple and isolatedalone, gene pathways contain multiple genes of which expressions could work in sequence to eventually accomplish complicated and integrated biological activities. Investigations focused on pathways have shed some light on potential roles of ‘pathway’ alterations on MDD development, however, most of them focused on pathophysiological rather than gene pathways, and there seems to have no previous in-depth studies targeting on gene pathways as a whole using deep sequencing in MDD patients or experimental animals. In order to better support genetic basic research and genome analysis, there have been bioinformatics tools for visualization, interpretation and analysis of gene pathways. REACTOME (13), together with KEGG (14) and other tools, have shown a high performance and have been widely used in gene pathway analysis and investigations.

Gene therapy refers to the introduction of exogenous normal genes into target cells to correct or compensate for diseases caused by defective and abnormal genes for therapeutic purposes. Gene therapy for targeting a variety of brain diseases, ranging from brain tumors and brain injury to dementia and motor neuron disease, has attracted significant interest from researchers (15) The development of suitable gene therapy regimens for specific genetic targets offers the possibility of treating MDD. Unfortunately, effective and safe systemic delivery of siRNA to the brain remains challenging due to biological barriers such as enzymatic degradation, short circulating lifetime, blood-brain barrier (BBB), inadequate tissue permeation, cellular endocytosis and cytoplasmic transport (16). With the rapid progression of nanotechnology, the combination strategies of nanotechnology with chemical and biological modification offer interesting potential to address these challenges in brain delivery of DNA, siRNA, miRNA and shRNA (17). Although there are currently few MDD therapeutic strategies based on nanotechnology coupled with gene therapy. As more MDD-related gene therapy targets emerge, MDD treatment strategies based on nanotechnology coupled with gene therapy will attract great interests.

Based on this knowledge, the current study was designed to identify gene pathway mutations in MDD patients and to investigate potential relationships between these pathway mutations and MDD development. The current research adopts a novel approach by focusing on the Digestion and Dietary Carbohydrate pathway, revealing a 100% mutation rate in MDD patients compared to matched healthy controls. This pathway’s unique association with MDD suggests a potential genetic mechanism contributing to the disorder’s development. By exploring gene pathways rather than individual polymorphisms, this study aims to provide a more holistic understanding of MDD, offering valuable insights for future diagnostic and therapeutic strategies. Novel drugs or novel drug delivery methods might arise according to the mutation mediated biological mechanism, such as the drug delivery platforms based on the mutated molecular targets or specific receptors. That can facilitate the transformation of the MDD gene screening results to the clinical application.

## 2 Materials and methods

### 2.1 Limitation and further investigation

Patients aged 18 ~ 60 years who were diagnosed as MDD using the Structured Clinical Interview for DSM-IV Disorders (SCID) were recruited consecutively at West China Hospital. Exclusion criteria included any history of psychosis, significant neurological or medical illness, currently receiving electroconvulsive therapy, and any history of alcohol or substance abuse or dependence. No relations of enrolled patients were included. This study was approved by the Clinical Trials and Biomedical Ethics Committee of Sichuan University. Written informed consents were obtained from all participants.

Healthy controls were recruited from the local area by advertisements and demographic characteristics including age and sex were matched with the depressed patients. All healthy control subjects were interviewed by experienced psychiatrists to ensure that no history of neuropsychiatric illness or brain injury and no known family history of depression or serious mental illness in first degree relatives existed.

### 2.2 Extra-deep whole genome sequencing and data processing

Genomic DNA was extracted from peripheral blood with TruSeq_DNA_SamplePrep kit (Illumina Inc., San Diego, CA, USA). Qubit 2.0 (Thermo Fisher Scientific Inc., Waltham, USA) was used to precisely quantify DNA concentrations. DNA samples with a content of 0.6μg or more were used and were fragmented to an average size of 180~280 bp to create a DNA library following established Illumina pairedend protocols (Illumina Inc.). The Agilent SureSelect Human All ExonV6 Kit (Agilent Technologies, Santa Clara, CA, USA) was used for exome capture. After the library was constructed, Qubit 2.0 was used for preliminary quantification, then Agilent 2100 (Agilent Technologies) was used to detect the insert size of the library. Illumina Novaseq 6000 platform (Illumina Inc.) was used for sequencing. The equations should be inserted in editable format from the equation editor.

### 2.3 Bioinformatics analysis process

After sequencing, base-call file conversion and demultiplexing were performed with the bcl2fastq software (Illumina). The resulting fastq data were analyzed by in-house quality control software to remove low quality reads, and were then aligned to the reference human genome (hs37d5) using the Burrows-Wheeler Aligner (BWA) (18), and duplicate readings were marked using Sambamba tools (19).

Single nucleotide variants (SNVs) and indels were called with GATK (3) to generate a gVCF file. Mutation detected with the recommended VQSR method to recalibrate and filter the quality values of the variant loci. GATK bundled real project data from omni,1000G,dbsnp,hapmap and mills were used for correction and filtering (https://gatk.zendesk.com/hc/en-us/articles/360035890811). The raw calls of SNVs and InDels were further filtered with the fol-lowing inclusion thresholds: 1) a read depth > 4; 2) a root-mean-square mapping quality of the covering reads > 30; 3) a variant quality score > 20.

Annotation was performed using ANNOVAR (June 8 2017) (4). Annotations included minor allele frequencies from the public control data sets as well as deleteriousness and conservation scores enabling further filtering and assessment of the likely pathogenicity of the variants.

Filtering of rare variants was performed as follows: (1) variants with an MAF less than 0.01 in 1000 genomic data (1000g_all) (5), esp6500siv2_all (6), gnomAD data (gnomAD_ALL and gnomAD_EAS) (7); (2) Only SNVs occurring in exons or splice sites (splicing junction 10 bp) were further analysed because we were interested in amino acid changes. (3) Synonymous SNVs that were not relevant to the amino acid alterna-tions predicted by dbscSNV were discarded; small fragment non-frameshift (<10 bp) indels in the repeat regions defined by RepeatMasker were also discarded. (4) Variations were screened according to their scores using SIFT (8), Polyphen (9), MutationTaster (10) and CADD (11) software programs. Potentially deleterious variations were retained if the score from more than half of these four software programs supported their potential harmfulness (12). Sites (> 2 bp) that did not affect alternative splicing were removed.

After these filtering process, gene enrichment analysis was adopted using Reactome database to identify the significant pathway covered by residual mutated genes in MDD and control groups.

### 2.4 Statistical analysis

Statistical comparisons were performed under SPSS 19.0 (SPSS, Inc., Chicago, USA). Age and gender were compared by Chi Square test respectively.

Pathway significant enrichment analysis was based on pathways in Reactome database (13), and a pathway would be considered mutation enrichment positive if at least one gene in this pathway turned out to be gene mutation enriched. Hypergeometric test was used to find pathways with significant enrichment in different expressed genes compared with the whole genomic background. The calculation formula was as follows:


$$p=1-\sum_{i=0}^{m-1}\frac{\left(\begin{array}{c} M\\ i \end{array}\right)\left(\begin{array}{l} N-M\\ n-i \end{array}\right)}{\left(\begin{array}{c} N\\ n \end{array}\right)}$$

N was the number of pathway-annotated genes in all genes;

n was the number of different expressed genes in N;

M was the number of genes annotated as a specific Pathway in all genes;

m was the number of different expressed genes annotated as a specific Path-way.

When p-value ≤ 0.05, differential genes were considered to be significantly enriched in this Pathway.

## 3 Results

### 3.1 Demographic and clinical characteristics

A total of 117 patients with MDD and 78 healthy controls were enrolled in this study. Age and gender were not significantly different between MDD patients and healthy controls. Demographic and clinical information for matched groups was pre-sented in **Table 1**.

**Table 1:** Demographic and clinical characteristics of MDD patients and healthy controls.

Characteristic	MDD		HC		*p* value
	Mean	SD	Mean	SD	
age, years	31.88	10.25	33.41	10.48	0.326
illness duration, months	6.62	6.48			
HAMD score	25.84	5.28			
Gender	N	%	N	%	*p* value
Female	75	64.1	46	59	0.47
Male	42	35.9	32	41	

MDD, major depressive disorder; HC, healthy control.

### 3.2 Summary of sequencing

**Figure 1:**
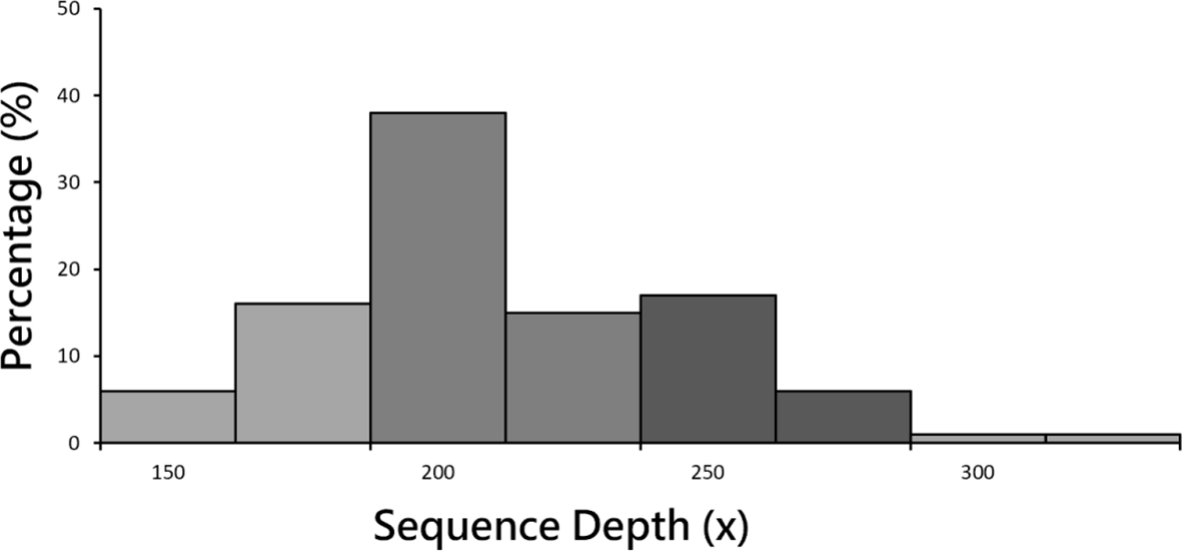
Sample sequencing depth distribution. Mean sequencing depth was 223x. Sequencing depth of 200 ~ 225 contained the highest rate (38.00%).

After the completion of sequencing, 78 healthy controls and 117 MDD patient groups underwent paired-end sequencing. Average sequencing depth was 223x (**Figure 1**), and sequencing depth of 200 ~ 225 contained the highest rate (38.00%). Average reads per sample was 44.12 million. Exon capture coverage rate was 99.9%, and average SNP number was 480,000. The data presented in the current manuscript are deposited in the zenodo repository, https://zenodo.org/, accession number is 7769978.

### 3.3 Mutations in digestion of dietary carbohydrate pathway

**Figure 2:**
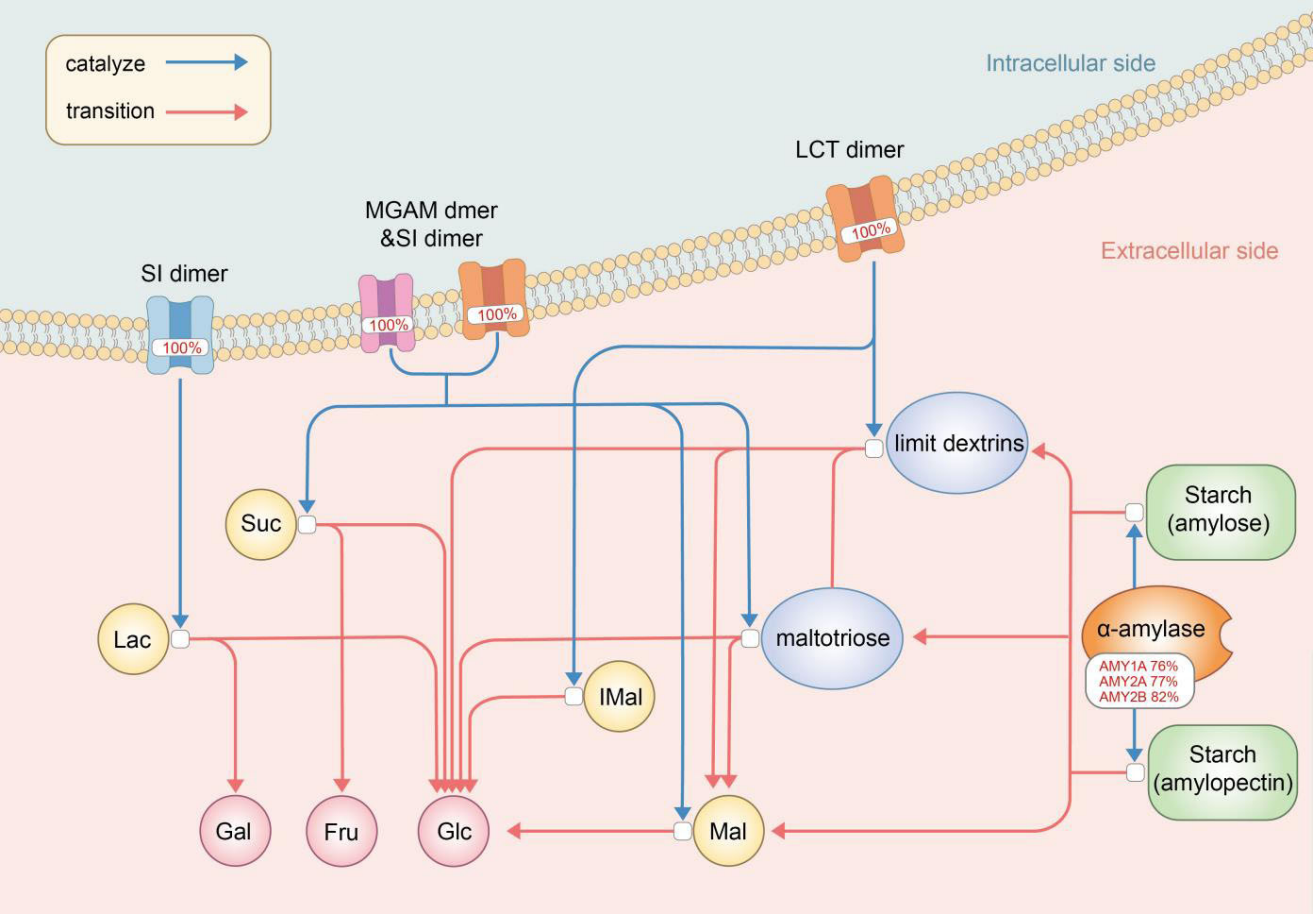
The digestion of dietary carbohydrate pathway (Carbohydrate pathway) with mutation rates. The digestion of carbohydrates begins with the action of amylase enzymes secreted in the saliva and small intestine, which convert it to maltotriose, maltose, limit dextrins, and some glucose. Digestion of the limit dextrins and disaccharides, both dietary and starch-derived, to monosaccharides - glucose, galactose, and fructose - is accomplished by enzymes located on the luminal surfaces of enterocytes lining the microvilli of the small intestine. AMY, amylase; SI, Sucrase-Isomaltase; MGAM, malt-as-glucoamylase; LCT, lactase; Mal, maltose; IMal, Suc, sucros; Lac, lactose; Glu, glucose; Fru, fructose; Gal, galactose.

**Figure 3:**
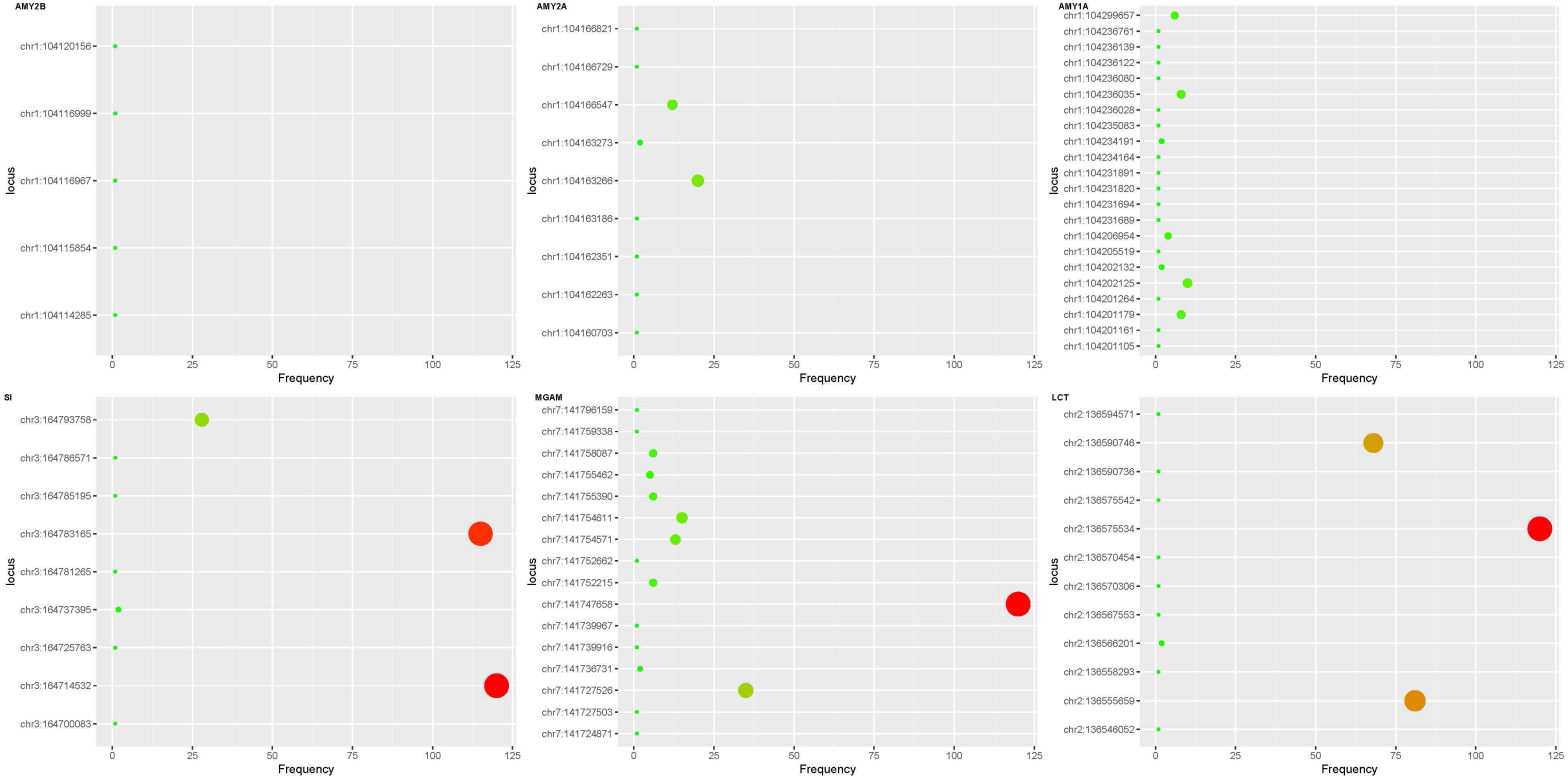
Exon mutations in digestion of dietary carbohydrate pathway. Mutations were found in all 6 gene exons in the pathway in the MDD patient group and none in the control group. The ordinate represents the coordinate of the mutation site on the chromosome, and the abscissa represents the frequency in the exon mutation of the sample. The AMY2B gene had fewer exon mutations than the AMY1A and AMY2A genes. Compared with SI, MGAM and LCT mutation sites of the three amylase genes were more dispersed. SI gene has 3 high frequency mutations (ch3:164793758, ch3:1647833165 and ch3:164725763), MGAM gene has 2 high-frequency mutations (ch7:141747658 and ch7:141727526), the LCT gene has 3 high frequency mutations (ch2:136590746, ch2:136575534 and ch2:136555659). AMY, amylase; SI, Sucrase-Isomaltase; MGAM, maltas-glucoamylase; LCT, lactase.

The Digestion of Dietary Carbohydrate pathway (Carbohydrate pathway, **Figure 2**) was revealed by gene enrichment analysis to contain 100% significant mutation gene enrichment (p-value<0.05) in MDD patients, which defined as at least 1 mutation in the 6 protein or protein complexes encoding genes composing the pathway, while 0 mutation gene enrichment happened in healthy controls (**Figure 3**). Mutation rates of the 6 composing genes in this pathway were 76.03% (amylase 1a), 76.86% (amylase 2a), 81.82% (amylase 2b), 100.00% (SI), 100.00% (MGAM) and 100.00% (LCT).

## 4 Discussion

Gene-involved mechanisms have long been a hotspot of MDD related investigations and it has been established that MDD is somehow inheritable (1, 20). However, despite that multiple studies have tried to identify specific candidate polymorphisms in single genes underlying MDD development, recent studies of multiple large samples have demonstrated that, there was no support for historical candidate gene to have any actual relationships with MDD development, including the most thoroughly investigated gene SLC6A4 (8), and common single-nucleotide polymorphisms (SNPs) account for only less than 30% MDD risk variance (21). Furthermore, the relationship between SNPs and MDD could be ambiguous, leading to more complicated interpretations of positive SNPs findings.

Deep/extra-deep sequencing has gradually become a mature method and has been increasingly used in latest studies. It allows better detections of rare or mixed mutations, when comparing to conventional sequencing, by sequencing a genomic region hundreds or even thousands of times (22, 23). Single gene analysis, which was commonly employed in genome/exon studies previously, does not consider the interaction between genes and ignores the effect produced by the coordination between genes in the biological process. On the contrary, gene enrichment analysis in the biological process identifies mutated genes by determining influences of mutations on a specific biological process or function. When mutated genes are significantly concentrated in a biological pathway, the mutant effects of these genes would have a significant impact on that pathway. Therefore, gene enrichment analysis could be used to reveal the effects of multiple gene mutations as a whole. The adoption of both deep sequencing and gene enrichment analysis together enables novel findings reported in the current study.

For the very first time, our study demonstrated a pathway, the Digestion and Dietary Carbohydrate pathway (Carbohydrate pathway), which has established gene compositions and specific genetic expressions with established functions, with a 100% gene mutations enrichment in MDD patients vs. 0 mutation enrichment in matched healthy controls.

### 4.1 Digestion of dietary carbohydrate pathway

The Digestion of Dietary Carbohydrate pathway (Carbohydrate pathway) contains genes encoding mediators from amylase enzymes in saliva and small intestine to sucrase-isomaltase (SI) dimers, maltas-glucoamylase (MGAM) dimers and lactase (LCT) dimers in small intestine, which all work as a whole to help carbohydrate digestion in the digestive tract. Each of these expressions is necessary and vital for one or several steps in the specific series of reactions which brings carbohydrate to be decomposed into glucose, galactose and fructose.

Association between sugar/carbohydrate intolerance, especially lactose and fructose malabsorption, and depression has long been recognized (24). Carbohydrate pathway contains genes encoding products involved in disaccharidases and monosaccharide metabolisms. Lactose is a kind of non-absorbable disaccharidases, and LCT mutations/polymorphisms in Carbohydrate pathway could result in incomplete hydrolysis of lactose into the monosaccharides, glucose and galactose (25). SI and MGAM take part in final steps of carbohydrate metabolism and SI is responsible for almost all sucrose activity (26). Fructose, a monosaccharide, demands SI dimers in this pathway to be generated from sucrose, and fructose malabsorption has been proved to be associated with depression (25), even though potential mechanism is unknown. Our results showed a 100% gene mutations enrichment of both LCT and SI dimers in MDD patients, which could render maldigested and malabsorbed lactose and fructose, leaving extra lactose and fructose retention in the small intestine. These abnormally retented lactose and fructose could interfere with tryptophan absorption, leading to an excessive formation of complexes of tryptophan, the precursor of serotonin. The resultant decreased serotonin synthesis compromises serotonergic neurotransmission, which is common in the central nervous system in major depression (27).

Recognition of the interaction between commensal bacteria in the colon, i.e. the gut microbiota, and central nervous system through the gut-brain axis prompts investigations focusing on the potential influence of gut microbiota on mental health, including MDD development (28), and it has been established that gut dysbiosis could somehow be associated with MDD (29, 30). Rodent studies have revealed that mice harboring ‘depression microbiota’ would come up with disturbances of gut microbiota and host metabolites involved in carbohydrate metabolism, and it was concluded that gut dysbiosis might somehow play a role in the development of depressive-like behaviors through a metabolism-mediated pathway (31). Congenital genetic mutations in Carbohydrate pathway targeting SI dimers could primarily affect digestive capacity of SI, while the maldigested carbohydrates could somehow modulate the gut microbiota, and probably the intestinal physiology (26), leading to a possible MDD attack.

One of the interesting findings in our study is the high mutation rates of genes encoding α-amylases including salivary amylases and intestinal amylases. Salivary α-amylase (sAA) activity was observed to be significantly decreased in both adult and adolescent MDD patients (32, 33). As sAA level changes in response to psychosocial stimulations and presents positive correlations with cardiovascular responses (34), it was frequently obtained as and only as an non-invasive indicator of stress response systems such as the hypothalamic-pituitary-adrenal (HPA) axis and sympathetic-adrenal-medullary (SAM) system, thus to better evaluate associations among stress, especially early life stress (ELS), HPA axis, SAM system and MDD (33–36). The high mutation rates of sAA genes found in our current study revealed a novel potential relationship between α-amylase on MDD development, that intestinal amylases and sAA might work directly by mutation-induced rather than HPA/SAM-induced alterations. This result might somehow lead to a direction of investigations involving α-amylases and MDD.

Another notable finding is that, multiple novel gene mutations were found only in MDD patients. The absence of documentation of these novel mutations in databases indicates that they have not been revealed or investigated before. Considering these mutations were established from actual clinical-diagnosed MDD patients, these findings provide huge potential for further basic and clinical drug R&D targets, which would help with MDD management greatly.

Based on the above relevant findings at the level of MDD gene mutations, it may be valuable to explore the sAA-related pathway as a target for MDD drug therapy and delivery. Recently, the emergence of RNA-based drug delivery systems for gene therapy has made treatment regarding established genetic targets possible. In particular, non-viral delivery systems, such as polymer-based or nanoliposome-based delivery systems, can bypass the limitations of viral delivery vectors by manipulating intracellular gene expression to produce therapeutic proteins or compensate for deficiencies due to mutations (reference). It might be the key to future mutation detection as well as gene therapy in MDD-related fields that designing RNA drugs or developing sAA-based prodrugs based on the above-mentioned mutant phenotypes or even individualized diagnostics would somehow modulate sAA levels by targeting the HPA axis, SAM axis and sAA-secreting gland cells, which are associated with MDD development, thus to modulate the stress response system to alleviate MDD symptoms.

Notably, not all genes in the Carbohydrate pathway showed a 100% mutation enrichment, e.g., Amylase 1a/1b mutations presented only in about 3/4 MDD patients. Meanwhile, even though LCT and SI mutations enrichment happened in 100% patients with MDD, former study has shown that primary-adult lactose malabsorption was not predictive for depressive symptoms (25). These results might somehow indicate the existence of influencing factors other than genetic abnormalities, which is consistent with a MDD heredity less than 100%. These factors might also play their roles together with mutated genes as well as independently. For example, gut microbiome status and stress development are clearly influenced by Carbohydrate pathway mutations and resultant abnormal gene expression products as mentioned above, they are also established to be under the reg-ulation of environmental factors at the same time. It might be reasonable to conclude that MDD development is the consequence of genetic and other factors including environmental ones.

In conclusion, this investigation represents a significant step toward unraveling the intricate genetic landscape of MDD, emphasizing the importance of gene pathways in the context of neurobiological perspectives. The identification of mutations within the Digestion and Dietary Carbohydrate pathway underscores the potential relevance of specific genetic mechanisms in MDD, paving the way for targeted interventions and personalized treatment approaches in the future.

### 4.2 Limitation and further investigation

Findings revealed in the current study enable a better understanding of gene pathways mutations status in MDD patients, revealing a possible genetic mechanism of MDD development and indicating a potential diagnostic or therapeutic target. However, it is still left uncertain that whether products of these mutated genes collaborate or work alone through different mechanisms to induce MDD, and how and to what extent that abnormal products by mutated gene expression could interfere with central nerve system and induce MDD. Environmental factors are also not included in this study. Further investigations are warranted to illustrate temporal and casual relationships between these pathways’ mutations and MDD and to develop related diagnosis or treatment strategies.

## Data availability statement

The data presented in the study are deposited in the zenodo repository, https://zenodo.org/, accession number 7769978.

## Ethics statement

The studies involving humans were approved by the Clinical Trials and Biomedical Ethics Committee of Sichuan University. The studies were conducted in accordance with the local legislation and institutional requirements. Written informed consent for participation in this study was provided by the participants’ legal guardians/next of kin.

## Author contributions

NZ: Writing – original draft, Writing – review & editing, Conceptualization, Data curation, Resources. JL: Data curation, Resources, Writing – review & editing. ZD: Resources, Writing – original draft, Data curation. YH: Data curation, Writing – review & editing. ZZ: Methodology, Writing – original draft. QG: Funding acquisition, Supervision, Writing – review & editing. WK: Funding acquisition, Resources, Supervision, Writing – review & editing.

## References

[1] Otte C Gold SM Penninx BW Pariante CM Etkin A Fava M. Major depressive disorder. Nat Rev Dis Primers. (2016) 2:16065. doi: 10.1038/nrdp.2016.65 27629598

[2] Lam RW McIntosh D Wang J Enns MW Kolivakis T Michalak EE. Canadian network for mood and anxiety treatments (CANMAT) 2016 clinical guidelines for the man-agement of adults with major depressive disorder: section 1. Disease burden and principles of care. Can J Psychiatry. (2016) 61:510–23. doi: 10.1177/0706743716659416 PMC4994789 27486151

[3] Malhi GS Mann JJ. Depression. Lancet. (2018) 392:2299–312. doi: 10.1016/S0140-6736(18)31948-2 30396512

[4] Greenberg PE Fournier AA Sisitsky T Pike CT Kessler RC. The economic burden of adults with major de-pressive disorder in the United States (2005 and 2010). J Clin Psychiatry. (2015) 76:155–62. doi: 10.4088/JCP.14m09298 25742202

[5] Sheehan DV Nakagome K Asami Y Pappadopulos EA Boucher M. Restoring function in major depressive dis-order: A systematic review. J Affect Disord. (2017) 215:299–313. doi: 10.1016/j.jad.2017.02.029 28364701

[6] Philibert RA Sandhu H Hollenbeck N Gunter T Adams W Madan A. The relationship of 5HTT (SLC6A4) methylation and genotype on mRNA expression and liability to major depression and alcohol dependence in subjects from the Iowa Adoption Studies. Am J Med Genet B Neuropsychiatr Genet. (2008) 147B:543–9. doi: 10.1002/ajmg.b.30657 PMC3643119 17987668

[7] Dong C Wong ML Licinio J. Sequence variations of ABCB1, SLC6A2, SLC6A3, SLC6A4, CREB1, CRHR1 and NTRK2: association with major depression and antidepressant response in Mexican-Americans. Mol Psychiatry. (2009) 14:1105–18. doi: 10.1038/mp.2009.92 PMC2834349 19844206

[8] Border R Johnson EC Evans LM Smolen A Berley N Sullivan PF. No support for historical candidate gene or candidate gene-by-interaction hypotheses for major de-pression across multiple large samples. Am J Psychiatry. (2019) 176:376–87. doi: 10.1176/appi.ajp.2018.18070881 PMC6548317 30845820

[9] Traks T Koido K Balotsev R Eller T Koks S Maron E. Polymorphisms of IKBKE gene are associated with major depressive disorder and panic disorder. Brain Behav. (2015) 5:e00314. doi: 10.1002/brb3.314 25798331 PMC4356867

[10] Rao S Yao Y Ryan J Li T Wang D Zheng C. Common variants in FKBP5 gene and major depressive disorder (MDD) susceptibility: a comprehensive me-ta-analysis. Sci Rep. (2016) 6:32687. doi: 10.1038/srep32687 27601205 PMC5013409

[11] Can MS Baykan H Baykan O Erensoy N Karlidere T. Vitamin D levels and vitamin D receptor gene polymor-phism in major depression. Psychiatr Danub. (2017) 29:179–85. doi: 10.24869/psyd.2017.179 28636576

[12] Liou YJ Chen TJ Tsai SJ Yu YW Chen SY Cheng CY. Evidence of involvement of the human Par-4 (PAWR) gene in major depressive disorder. World J Biol Psy-chiatry. (2011) 12:288–95. doi: 10.3109/15622975.2010.509451 20735158

[13] Fabregat A Sidiropoulos K Viteri G Forner O Marin-Garcia P Arnau V. Reactome pathway analysis: a high-performance in-memory approach. BMC Bioinf. (2017) 18:142. doi: 10.1186/s12859-017-1559-2 PMC5333408 28249561

[14] Kanehisa M Sato Y Kawashima M Furumichi M Tanabe M. KEGG as a reference resource for gene and protein annotation. Nucleic Acids Res. (2016) 44:D457–462. doi: 10.1093/nar/gkv1070 PMC4702792 26476454

[15] Yan L Yang Y Zhang W Chen X. Advanced materials and nanotechnology for drug delivery. Adv Mater. (2014) 26:5533–40. doi: 10.1002/adma.201305683 24449177

[16] Miao YB Zhao W Renchi G Gong Y Shi Y. Customizing delivery nano-vehicles for precise brain tumor therapy. J Nanobiotechnol. (2023) 21:32. doi: 10.1186/s12951-023-01775-9 PMC9883977 36707835

[17] Mischoulon D Hylek L Yeung AS Clain AJ Baer L Cusin C. Corrigendum to “Randomized, proof-of-concept trial of low dose naltrexone for patients with breakthrough symptoms of major depressive disorder on antidepressants” [J. Affect. Disord. 208 (2017, Jan. 15) 6-14, doi: 10.1016/j.jad.2016.08.029, Epub 2016 Oct. 1]. J Affect Disord. (2018) 227:198. doi: 10.1016/j.jad.2016.08.029 29100152

[18] Li H Durbin R. Fast and accurate short read alignment with Burrows-Wheeler transform. Bioinformatics. (2009) 25:1754–60. doi: 10.1093/bioinformatics/btp324 PMC2705234 19451168

[19] Tarasov A Vilella AJ Cuppen E Nijman IJ Prins P. Sambamba: fast processing of NGS alignment formats. Bio-informatics. (2015) 31:2032–4. doi: 10.1093/bioinformatics/btv098 PMC4765878 25697820

[20] Sullivan PF Neale MC Kendler KS. Genetic epidemiology of major depression: review and meta-analysis. Am J Psychiatry. (2000) 157:1552–62. doi: 10.1176/appi.ajp.157.10.1552 11007705

[21] Peterson RE Cai N Bigdeli TB Li Y Reimers M Nikulova A. The genetic architecture of major depressive disorder in Han Chinese women. JAMA Psychiatry. (2017) 74:162–8. doi: 10.1001/jamapsychiatry.2016.3578 PMC5319866 28002544

[22] Mirebrahim H Close TJ Lonardi S. *De novo* meta-assembly of ultra-deep sequencing data. Bioinformatics. (2015) 31:i9–16. doi: 10.1093/bioinformatics/btv226 26072514 PMC4765875

[23] Ajay SS Parker SC Abaan HO Fajardo KV Margulies EH. Accurate and comprehensive sequencing of personal genomes. Genome Res. (2011) 21:1498–505. doi: 10.1101/gr.123638.111 PMC3166834 21771779

[24] Varea V de Carpi JM Puig C Alda JA Camacho E Ormazabal A. Malabsorption of carbohydrates and depression in children and adolescents. J Pediatr Gastroenterol Nutr. (2005) 40:561–5. doi: 10.1097/01.mpg.0000153005.61234.28 15861016

[25] Enko D Wagner H Kriegshauser G Brandmayr W Halwachs-Baumann G Schnedl WJ. Assessment of tryptophan metabolism and signs of depression in individuals with carbohydrate malabsorp-tion. Psychiatry Res. (2018) 262:595–9. doi: 10.1016/j.psychres.2017.09.049 28965810

[26] Gericke B Amiri M Naim HY. The multiple roles of sucrase-isomaltase in the intestinal physiology. Mol Cell Pediatr. (2016) 3:2. doi: 10.1186/s40348-016-0033-y 26812950 PMC4728165

[27] Lopresti AL Hood SD Drummond PD. A review of lifestyle factors that contribute to important pathways associated with major depression: diet, sleep and exercise. J Affect Disord. (2013) 148:12–27. doi: 10.1016/j.jad.2013.01.014 23415826

[28] Peirce JM Alvina K. The role of inflammation and the gut microbiome in depression and anxiety. J Neurosci Res. (2019) 97:1223–41. doi: 10.1002/jnr.24476 31144383

[29] Sharon G Sampson TR Geschwind DH Mazmanian SK. The central nervous system and the gut microbiome. Cell. (2016) 167:915–32. doi: 10.1016/j.cell.2016.10.027 PMC5127403 27814521

[30] Jiang H Ling Z Zhang Y Mao H Ma Z Yin Y. Altered fecal microbiota composition in patients with major depressive disorder. Brain Behav Immun. (2015) 48:186–94. doi: 10.1016/j.bbi.2015.03.016 25882912

[31] Zheng P Zeng B Zhou C Liu M Fang Z Xu X. Gut microbiome remodeling induces depressive-like behaviors through a pathway mediated by the host’s metabolism. Mol Psychiatry. (2016) 21:786–96. doi: 10.1038/mp.2016.44 27067014

[32] Szarmach J Cubala WJ Landowski J Chrzanowska A. No relationship between baseline salivary alpha-amylase and State-Trait Anxiety Inventory Score in drug-naive patients with short-illness-duration first episode major depressive disorder: An exploratory study. J Clin Exp Dent. (2017) 9:e527–30. doi: 10.4317/jced.53631 PMC5410672 28469817

[33] Jezova D Trebaticka J Buzgoova K Durackova Z Hlavacova N. Lower activity of salivary alpha-amylase in youths with depression. Stress. (2020) 23:688–93. doi: 10.1080/10253890.2020.1777975 32510266

[34] Mielock AS Morris MC Rao U. Patterns of cortisol and alpha-amylase reactivity to psychosocial stress in maltreated women. J Affect Disord. (2017) 209:46–52. doi: 10.1016/j.jad.2016.11.009 27875756 PMC5191933

[35] Booij SH Bos EH Bouwmans ME van Faassen M Kema IP Oldehinkel AJ. Cortisol and alpha-Amylase Secretion Patterns between and within Depressed and Non-Depressed Indi-viduals. PloS One. (2015) 10:e0131002. doi: 10.1371/journal.pone.0131002 26148294 PMC4492984

[36] Inoue A Oshita H Maruyama Y Tanaka Y Ishitobi Y Kawano A. Gender determines cortisol and alpha-amylase responses to acute physical and psychosocial stress in patients with borderline personality disorder. Psychiatry Res. (2015) 228:46–52. doi: 10.1016/j.psychres.2015.04.008 25979467

